# Correction: One-pot synthesis of a new generation of hybrid bisphosphonate polyoxometalate gold nanoparticles as antibiofilm agents

**DOI:** 10.1039/c9na90050k

**Published:** 2019-09-18

**Authors:** S. Tomane, E. López-Maya, S. Boujday, V. Humblot, J. Marrot, N. Rabasso, J. Castells-Gil, C. Sicard, A. Dolbecq, P. Mialane, A. Vallée

**Affiliations:** Institut Lavoisier de Versailles, UVSQ, UMR CNRS 8180, Université Paris-Saclay 45, avenue des Etats-Unis 78035 Versailles Cedex France anne.vallee@uvsq.fr; Sorbonne Université, Laboratoire de Réactivité de Surface (LRS), UMR CNRS 7197 4 place Jussieu 75252 Paris France; Institut de Chimie Moléculaire et des Matériaux d’Orsay, UMR CNRS 8182, Université Paris-Sud, Université Paris Saclay 15 rue Georges Clemenceau 91405 Orsay Cedex France; Universidad de Valencia (ICMol) Catedrático José Beltrán-2 46980 Paterna Spain

## Abstract

Correction for ‘One-pot synthesis of a new generation of hybrid bisphosphonate polyoxometalate gold nanoparticles as antibiofilm agents’ by S. Tomane *et al.*, *Nanoscale Adv.*, 2019, **1**, 3400–3405.

The authors regret the omission of one affiliation for one of the authors, Somia Tomane, from the original manuscript. The corrected list of authors and affiliations for this paper is as shown above.

The Royal Society of Chemistry apologises for these errors and any consequent inconvenience to authors and readers.

## Supplementary Material

